# Components of variance in transcriptomics based on electrophoretic separation of cDNA fragments (cDNA-AFLP)

**DOI:** 10.1002/elps.200800756

**Published:** 2009-07

**Authors:** Arne Weiberg, Petr Karlovsky

**Affiliations:** Molecular Phytopathology and Mycotoxin Research Unit, University of GoettingenGoettingen, Germany

**Keywords:** Band matching, cDNA-AFLP, Peak matching, Peak recognition, Variance components

## Abstract

The sources of variance and errors in transcriptomics based on the electrophoretic separation of amplified cDNA fragments were investigated using cDNA-amplified fragment length polymorphism (AFLP). Transcriptome profiles of the plant-pathogenic fungus *Verticillium longisporum* were generated by a standard cDNA-AFLP protocol followed by electrophoretic separation of amplified DNA fragments in flatbed polyacrylamide gels with fluorescence detection as well as by capillary electrophoresis (DNA sequencer). The total variance was partitioned into contributions of cDNA synthesis, adapter ligation, preamplification, amplification, and electrophoresis. Parameters of computer-aided peak recognition and matching were investigated and strategies improving matching success based on double passage with different signal intensity thresholds were developed. The overall quality of data was similar for cDNA-AFLP and microarray hybridization. Variance of cDNA-AFLP was independent of signal intensity, whereas microarray data showed higher variance for low-intensity signals. Capillary electrophoresis significantly reduced the number of wrongly matched and unmatched signals as compared with flatbed gels. These results are also likely to apply to related electrophoresis-based transcriptome analysis techniques such as mRNA differential display.

## 1 Introduction

The set of abundances of mRNA molecules in an organ, tissue, or microbial culture represents a snapshot of gene expression at the transcriptional level. Simultaneous analysis of these mRNA molecules, designated transcriptomics, is a fundamental concept of functional genomics, which seeks to unravel the roles of individual genes in biological functions and processes. As gene expression is regulated primarily at the transcription level, comparison of the transcriptome state under different physiological or developmental stages reveals stage-specific patterns of gene expression and facilitates the assignment of biological functions to genes.

So-called close-end transcriptomic techniques, most prominent among them being microarray hybridization, require prior knowledge of gene sequences and are therefore unsuitable for organisms with limited availability of sequence data. Open-end techniques do not require prior sequence knowledge and can therefore be used as gene discovery tools. Among the latter methods, electrophoretic analysis of cDNA fragments amplified by randomly primed PCR (mRNA differential display 1) or by PCR primed at oligonucleotide adapters attached to DNA by ligation (cDNA-amplified fragment length polymorphism (AFLP)) has gained the most popularity.

cDNA-AFLP is based on selective amplification of subsets of restriction fragments originating from double-stranded DNA complementary to the transcriptome. cDNA is digested with two restriction endonucleases, resulting fragments are ligated to DNA adapters and amplified by PCR with adapter-specific primers. Subsets of these fragments are then amplified with primers, which consist of sequences complementary to the adapters and of additional, so-called selective nucleotides at the 3′ terminus. For all combinations of *N* selective nucleotides, DNA fragments are partitioned into 4*^N^* subsets, which are separately amplified and analyzed by electrophoresis 2. A recent innovation of the protocol consists or the elimination of redundancy, known as the “one gene–one tag” protocol 3,4.

Both mRNA differential display and cDNA-AFLP can be used for organisms of all kingdoms. cDNA-AFLP is reported to be superior to mRNA differential display because of its higher reproducibility and lower number of false positives 5. Industrial high-throughput transcriptome analysis systems derived from or inspired by cDNA-AFLP have also been highly successful 6. cDNA fragments revealing differences in expression under relevant conditions usually must be sequenced, but when a database of cDNA sequences or the genome sequence is available, cDNA-AFLP fragments can be matched to genes based on their size and flanking regions, which consist of recognition sites for restriction endonucleases extended by one to four selective nucleotides 7,8.

Visualization of DNA fragments in cDNA-AFLP and mRNA differential display protocols was originally achieved by using radioactively labeled primers 1,2 and later replaced by the incorporation of radioactively labeled nucleotides during PCR 9. With the widespread availability of DNA sequencers, labeling cDNA-AFLP and mRNA differential display products with fluorescent dyes became common. Data processing in electrophoresis-based transcriptomics consists of five steps: band or peak recognition, adjustment of mobilities among lanes or capillaries, signal matching, normalization of intensities, and comparative analysis. Although capillary electrophoresis is amenable to automation and offers higher throughput, flatbed polyacrylamide gels are still used because they allow DNA from bands of interest to be extracted from the matrix for cloning or sequencing. Although band matching within a single flatbed gel is possible, calibration standards are needed for comparisons among gels 10,11. Anonymous mobility standards *(e.g.* cDNA-AFLP products of a single DNA sample loaded onto all gels to be compared) are in principle sufficient for mobility adjustments, but using standards of known length greatly increases the value of the data set, because they facilitate the matching of bands to genes without the need to determine their sequences experimentally 7. In capillary electrophoresis, size standards are obligatory because differences in the electrophoretic behavior among capillaries may be large. A specific feature of capillary electrophoresis is that samples analyzed in the same capillary must be labeled with different fluorescent dyes, which usually affect electrophoretic behavior differently. For correct peak matching, these differences have to be compensated for. Hierarchical linkage clustering was suggested as a means of improving peak matching in cDNA-AFLP with fragment separation on a capillary sequencer 12.

Variance and statistical error are central to data processing in transcriptomics. In microarray hybridization, variance has been thoroughly studied and partitioned into components assigned to single experimental steps 13,14. This analysis has been lacking for electrophoresis-based transcriptomics, though cDNA-AFLP, mRNA differential display, and related techniques have been increasingly used for quantitative transcriptome analysis. Systematic errors in electrophoresis-based transcriptomics can be excluded by experimental design and normalization, but variance introduced at different experimental steps is unavoidable, and its effect on data quality is poorly understood. Apart from statistical errors, missing and wrong assignments made during signal matching may seriously impair the results of electrophoresis-based transcriptomics. These errors are specific to cDNA-AFLP and related techniques because they do not occur in microarray hybridization and sequencing-based transcriptome analysis.

In this work we studied the source of errors in cDNA-AFLP. The total variance was partitioned into the contributions of individual steps, the effect of position tolerance (PT) on the number of missing and wrong band assignments was investigated and data processing strategies for the minimization of these errors were developed.

## 2 Materials and methods

### 2.1 Fungal cultures and RNA extraction

*Verticillium longisporum* isolate 43 was maintained as described previously 15. Cultures for RNA isolation were grown in 5 ml SXM liquid medium 16 stationary cultures at 23°C and a 12 h day/night cycle; to start the culture, the medium was inoculated with 10 μL of 10^6^ spores/mL of a glycerin spore suspension. Mycelium was crushed under liquid nitrogen, and total RNA was extracted using a guanidinium isothiocyanate protocol with LiCl precipitation 17.

### 2.2 cDNA-AFLP protocol

**Figure 1:**
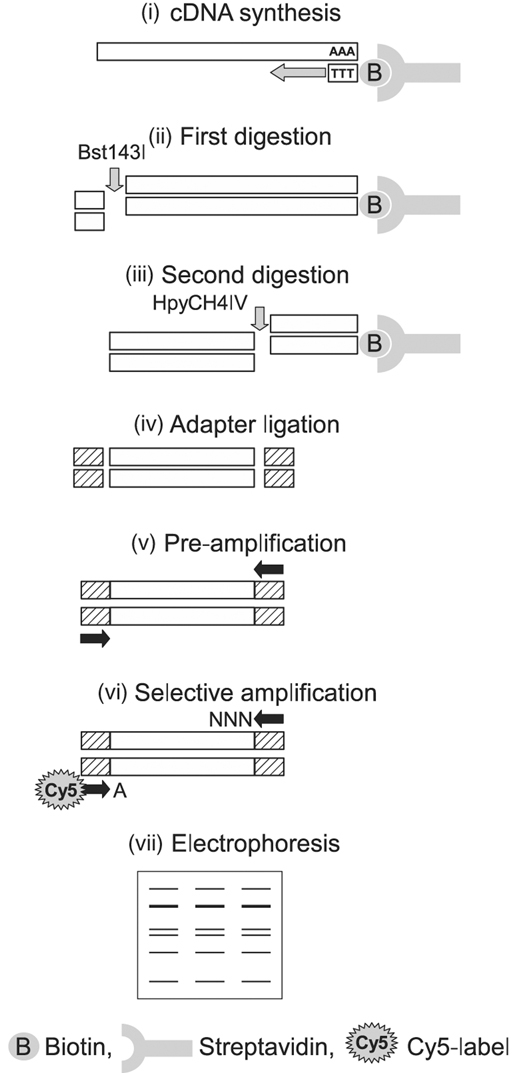
Workflow of cDNA-AFLP. Outline of the cDNA-AFLP procedure using the Bst143I and HpyCH4IV restriction enzyme combination: (i) mRNA is converted into double-stranded cDNA using a biotinylated oligo-dT primer. cDNA molecules are immobilized *via* the biotin tag to a streptavidin-coated reaction tube. (ii) First digestion of cDNA with Bst143I (indicated by gray arrow) and washing off the mobilized cDNA fragments. (iii) Second digestion of immobilized cDNA fragments with HpyCH4IV (indicated by gray arrow) and collection of mobilized cDNA fragments. These are used as template for (iv) ligation of DNA adapter to the restriction sites overhang. (v) Non-selective PCR amplification of cDNA fragments using primers (indicated by black arrows) compatible to adapter sequences. (vi) Final selective amplification of subsets of cDNA fragments using Bst143I+A and HpyCH4IV+NNN primers representing four selective nucleotides, with the Bst143I primer being labeled with the fluorescent dye Cy5 to allow subsequent detection of the cDNA fragments; and (vii) electrophoretic size fractionation and display on denaturing polyacrylamide gels of the Bst143I/HpyCH4IV cDNA fragments.

Total RNA was used for cDNA-AFLP analysis according to Bachem Oligonucleotides 2, modified by capturing mRNA on streptavidin-coated PCR tubes (Roche Applied Science, Penzberg, Germany) in combination with a dT_20_ primer labeled with biotin at its 5′ terminus for the immobilization of transcript molecules during cDNA synthesis 18,19. A schematic workflow of cDNA-AFLP procedure is shown in Fig. 1. For first strand cDNA synthesis 1–5 μg of total RNA was mixed with 50 pmol biotinylated dT_20_ primer, 2.5 mM dNTP mix, 40 U RNAse inhibitor (Fermentas, St. Leon-Roth, Germany) and 300 U RevertAid H-minus reverse transcriptase (Fermentas) within a streptavidin-coated reaction tube in a total volume of 50 μL and incubated for 1 h at 42°C. After incubation 40 μL of the reaction volume was discarded. Second strand of cDNA was synthesized within the strepatavidin-coated tube by adding 7.5 nmol of each dNTP 0.75 U T4 DNA Ligase (Fermentas), 3.75 U *Escherichia coli* DNA polymerase I (Fermentas), 1.12 U RNA nuclease H (Fermentas), in 60 μl water to the retained 10 μL of first strand synthesis reaction volume to obtain a total volume of 60 μL 18. Immobilized cDNA was digested by restriction endonucleases as follows: cDNA was first digested with restriction enzyme Bst143I (isoschisomer of MboI, purchased from Fermentas), and released cDNA fragments were washed from the column. This step, known as the “one gene–one tag” modification, reduces the redundancy 3,4. Truncated cDNA template bound to the column was digested with restriction endonuclease HpyCH4IV (isoschisomer of MaeII, New England Biolabs, Beverly, MA, USA). Adapters were ligated to the mobilized cDNA fragments and PCR was performed according to Bachem *et al*. 2. The sequences of the adapters and primers used in this work are listed in Table 1. The 5′ terminus of the Bst143I amplification primer was labeled with fluorescent dye Cy5 (Amersham Biosciences, Piscataway, NJ, USA).

**Table 1:** Oligonucleotides

Restriction site	Purpose	Sequence
Bst143I	Adapter	GATCCCTGCAGGGTCGTGCTAGTAGCT
		TACAGCTACTAGCACGACCCTGCAGG
	Preamplification primer	AGCTACTAGCACGACCCTGCA
	Amplification primer	Cy5-ACCCTGGGGATCA
HpyCH4IV	Adapter	CAACGTCACACTAACACTGAGCGGCCGC
		CGGCGGCCGCTCAGTGTTAGTGTGACG
	Preamplification primer	CGTCACACTAACACTACG
	Amplification primer	AGCGGCCGCCGTNNNa)

a) The extensions of HpyCH4IVI amplification primer were ACG, TTA, and CGT.

### 2.3 Data recording

Cy5-labeled cDNA fragments were separated and recorded on two different automated DNA sequencers, the capillary-based sequencer CEQ 8000 (Beckman Coulter, Fullerton, CA, USA) and flatbed polyacrylamide gel sequencer ALFExpress II (Amersham Biosciences). The conditions for the separation on CEQ 8000 were 4.0 kV and 10 μA for 45 min at 50°C, with capillaries of 33 cm long (inlet to outlet: 33 cm; inlet to detector: 30 cm) and 75 μm diameters using linear polyacrylamide separation matrix LPA-1 (Part no. 608105; the company did not reveal the concentration of linear polyacrylamide in the matrix). The electrophoresis on ALFExpress II was performed at 1.5 kV and 60 mA for 700 min on a 7% polyacrylamide gel (acrylamide/bisacrylamide ratio 19:1) ReproGel™ LongRead (Amersham Biosciences), of 0.3 mm thickness; the distance from inlet to outlet was 250 mm, distance from inlet to detector was 200 mm. Electropherograms were exported as CRV-files from CEQ 8000, and ALX-files generated by ALFExpress II were converted into TIFF files by the ALFwin™ Sequence Analyser 2.00 software (Amersham Biosciences). CRV and TIFF files were imported into GelCompar II software package version 4.0 (Applied Maths, Sint-Martens-Latem, Belgium) for quantitative analysis.

### 2.4 Data processing

CRV files from the capillary sequencer were converted into virtual pseudo gels by GelCompar II. Data from both sequencers were analyzed in the same way according to protocol consisting of gel-lane definition, background subtraction, averaging, noise reduction, spike removal and smoothing, mobility adjustment, band or peak detection, signal matching, and export of the results into a spreadsheet. Intensity thresholds for automated peak detection was set to 1% relative to the highest signal intensity detected in the same lane, except when noted otherwise. Automated signal matching was performed with a PT of 0.3%, except when noted otherwise. This corresponded to 1-bp-tolerance for fragments of up to 300 bp. The results of signal matching were checked visually. The heights of densitometry peaks corresponding to virtual bands were determined and exported into a spreadsheet.

### 2.5 Normalization of signal intensity values

Normalization of intensity values is required in comparative transcriptomics to compensate for differences in sample loading, pipetting errors, varying efficiencies of labeling, and other experimental factors. The crux of the normalization is that signals affected by the treatment have to be excluded from the calculation of a normalization factor, but these signals can only be recognized after normalization. The problem is solved by an algorithm that identifies induced/suppressed signals based on a comparison of ratios of signal intensities in treated samples and controls; the algorithm excludes these signals from the calculation of the normalization factor 20. Although no truly suppressed or induced transcripts were expected to occur in this work, the normalization algorithm was applied to match the conditions under which cDNA-AFLP is normally used.

### 2.6 *MA* plots

Scatter graphs (*MA* plots) show binary logarithm of the geometrical mean of paired and normalized cDNA signal intensities as function of signal intensities according to Dudoit *et al*. 21. *M* and *A* are defined as follows: 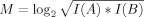 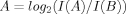 *I*(*A*) and *I*(*B*) are intensities of paired signals (matching cDNA-AFLP bands).

## 3 Results and discussion

### 3.1 Variance of cDNA-AFLP data: Comparison with microarray hybridization

The variance of raw data in both electrophoresis- and microarray hybridization-based transcriptomics is too high to allow for reliable quantitative interpretation of a single experiment, though qualitative evaluations of data from single experiments with the aim of discovering candidate genes have often been published. For quantitative evaluation, the use of replicates, internal controls, and adequate statistical treatment is essential. The largest contribution to variance in cDNA-AFLP originates from the biological variation inherent in the sample. Its size depends on the nature of the sample; for instance, mRNA levels may be expected to vary more in field samples than in samples from organisms grown under controlled conditions because microclimate, infection with pathogens, and other factors are more likely to affect gene expression in the field than in a growth chamber.

As a model for the analysis of variance in electrophoresis-based transcriptomics, we used cDNA-AFLP data generated with replicated cultures of the plant-pathogenic fungus *V. longisporum*. As the cultures were started from defined spore suspensions and growth conditions were strictly controlled, we expected the biological variance to fall close to the lower end of what is typically encountered in transcriptomics.

**Figure 2:**
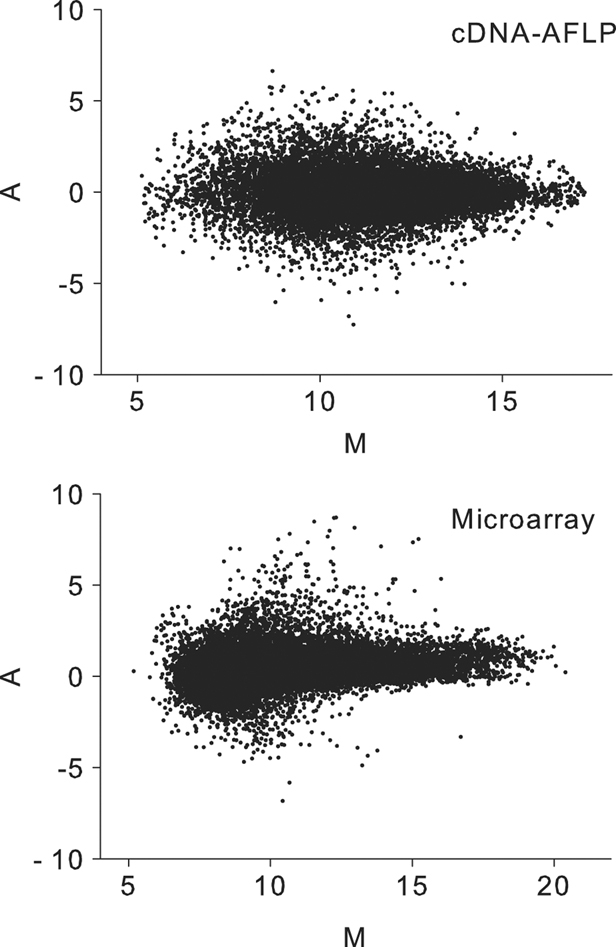
Comparison of cDNA-AFLP and microarray hybridization. Scatter plots of cDNA-AFLP-based (upper part) and microarray hybridization-based (lower part) data for biological replicates of identical samples are shown, each set comprising 11 233 data points. cDNA data were obtained from two *V. longisporum* cultures grown axenically in xylem-simulating medium 16. Microarray data originated from two commercial human reference RNAs (Universal Human Reference RNA from Stratagene and a Human Brain Reference RNA from Ambion); the data were downloaded from Microarray Quality Control web site (http://edkb.fda.gov/MAQC/MainStudy/). On horizontal axis binary logarithm of the geometrical mean of intensities of paired, normalized cDNA signals are shown. Vertical axis shows binary logarithm of intensity ratios of paired signals.

**Figure 3:**
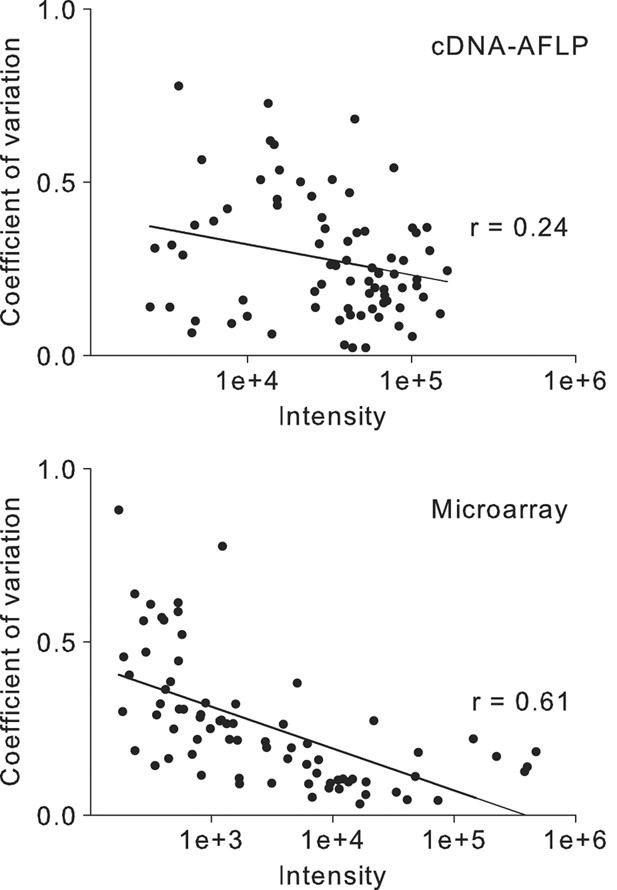
Variance and signal intensity in cDNA-AFLP and microarray hybridization. CV for technical triplicates was plotted against the geometric mean of signal intensity. “*r*” stands for the Pearson product–moment correlation coefficient.

**Figure 4:**
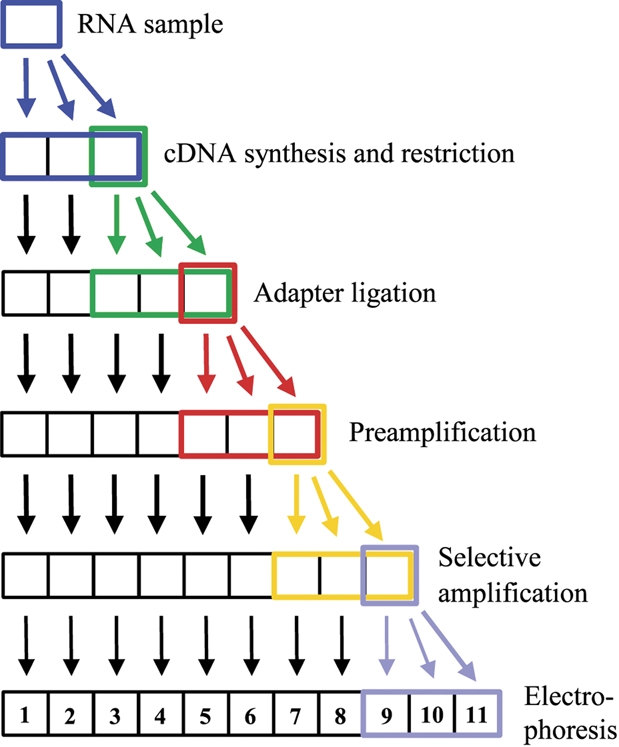
Sampling for the determination of partition of variance. *A* hierarchically ordered triplicate scheme was used for the estimation of the contribution of individual steps to total variance. Starting from a single RNA sample, one product was randomly selected at each step (this sample was placed at the right-most position in the scheme) and used to generate three replicates. The selected sample and the resulting replicas are labeled with the same color. Cumulative variance of all steps from “*n*” to 5 was estimated as the variance of cDNA-AFLP patterns resulting from replicas generated at step “*n*”.

Comparison of DNA-AFLP data with a typical microarray experiment is shown in Fig. 2 as MA plots, in which the binary logarithm of ratio of intensities for each signal is plotted against the geometrical mean of intensities. Microarray hybridization data were downloaded from the Microarray Quality Control (MAQC) web site (http://edkb.fda.gov/MAQC/MainStudy/). These data were obtained with commercially available RNA (Universal Human Reference RNA [UHRR] from Stratagene and Human Brain Reference RNA [HBRR] from Ambion) using Agilent one-color whole-genome human microarray 22. Visual inspection of scatter plots revealed that variance of microarray data declined with signal intensity. This is typical for microarray data (*e.g.* see Fig. 4 in 14, Fig. 1 in 23, and Fig. 2 in 24). No such relationship was apparent neither in cDNA-AFLP data generated in this work (Fig. 2) nor in published data generated with a related protocol (Fig. 3 in 25). Discarding data for targets with low-signal intensity is a common practice aimed at improving data quality in microarray hybridization 22, though it sacrifices valuable information.

In the next experiment, we compared data from three technical replicates, each generated from a single RNA preparation. As in the previous comparison, RNA from the *V. longisporum* culture was used for cDNA-AFLP, and microarray hybridization data for commercial human RNA (25% UHRR: 75% HBRR mixture) were downloaded from the MAQC web site (http://edkb.fda.gov/MAQC/MainStudy/). Microarray data were reduced to 81 randomly selected data points to obtain a data set of equal size as the cDNA-AFLP set. As only 94% of cDNA-AFLP signals could be matched in all three samples, we removed 6% of data points with the lowest mean intensities from the microarray data set.

For each set of technical replicates, the geometric mean of signal intensities was plotted against the CV (relative standard deviation) (Fig. 3). The analysis confirmed that the variance of cDNA-AFLP was independent of signal intensity (Pearson product–moment correlation coefficient *r*=0.24, correlation not significant at *p*=0.01). The variance of microarray data increased with declining signal intensity (*r*=0.61, significant at *p*<1E−06, normality confirmed by Kolmogorow–Smirnow test).

The mean values of the coefficients of variation were determined as follows: CV was calculated for each three matched bands in technical triplicates for both cDNA-AFLP experiment and microarray hybridization data. Mean values of CVs for cDNA-AFLP and microarray data were 26 and 21%, respectively, indicating a slightly better overall reproducibility of the latter technology.

### 3.2 Components of variance

Understanding how different steps contribute to the total variance of cDNA-AFLP will facilitate the identification of procedures that should be optimized. As the variance caused by individual steps except electrophoretic separation was not directly accessible, we generated triplicates in each step of the protocol in a hierarchical manner (Fig. 4). Total variance caused by all steps beginning with the replicates up to the final electrophoretic step was then determined. The contribution of the *n*-th step to variance was estimated as the difference between the variance of an experiment in which the *n*-th step was replicated and the variance of an experiment in which the (*n*+1)-th step was replicated (error propagation). For example, analysis of samples 5, 6, and 7 (Fig. 4) would provide the joint contribution of preamplification, amplification, and electrophoresis to the total variance of a cDNA-AFLP experiment. The variance of the final electrophoretic step was determined directly by repeating the separation of a single cDNA-AFLP product.

Because of the widespread availability of DNA sequencers, electrophoretic separation of fragments generated by cDNA-AFLP analysis has shifted from flat polyacrylamide gels to capillary electrophoresis. To determine how this shift affects the variance in transcript profiling, we analyzed data sets generated both with the help of ALFExpress II (GE Health Care, formerly Amersham Biotech), which is a flat gel-based automated DNA sequencer, and with CEQ 8000 (Beckman Coulter), which is a DNA sequencer based on capillary electrophoresis.

**Figure 5:**
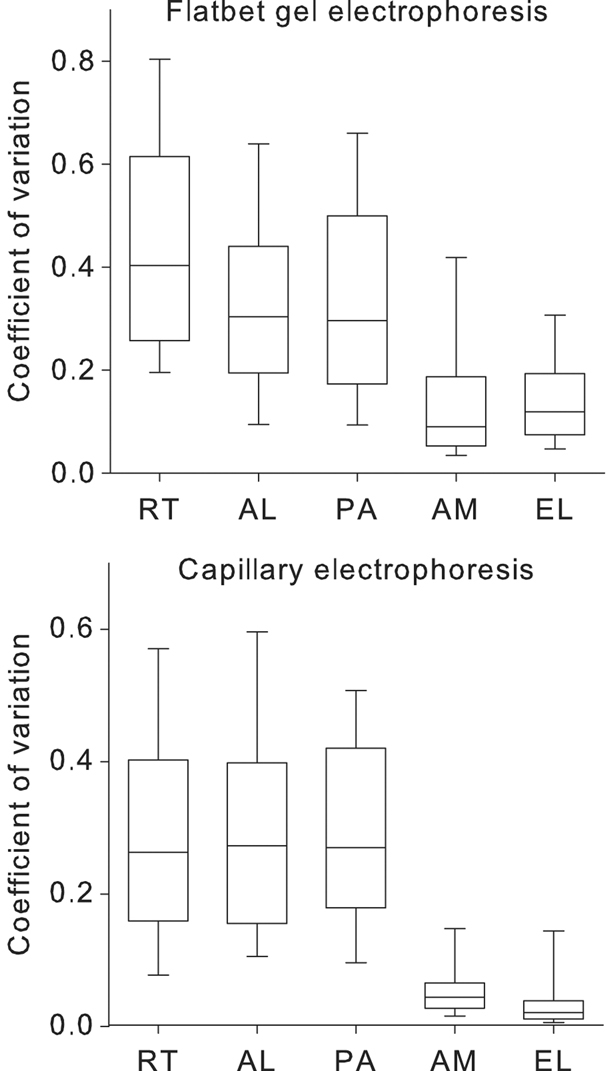
Contribution of experimental steps to variance in cDNA-AFLP. Triplicate samples were analyzed using flatbet gel electrophoresis (ALFExpress II; upper part) and capillary electrophoresis (CEQ 8000; lower part). Interquartile ranges (boxes), medians (horizontal bar within the boxes), and tenth and 90th percentile values (whiskers) for the CV are shown. The contribution of step “*n*” to the variance corresponds to the difference between the variance of replicates in step “*n*” and step “*n*+1”. RT, cDNA synthesis and restriction; AL, adapter ligation; PA, preamplification; AM, selective amplification; EL, electrophoresis.

The results of the dissection of variance components are shown in Fig. 5. As expected, the total variance grew as the step-generating replicates, and the contribution of different steps to variance varied. The largest contribution stemmed from preamplification and cDNA synthesis. It is likely that these two steps are also the major source of variation in related electrophoresis-based transcriptomics techniques such as mRNA differential display and GeneCalling 6. These steps should be targeted for optimization when reproducibility is an issue. The reproducibility of capillary electrophoresis was significantly better than that of flatbed electrophoresis (median CVs of 2 *versus* 12%). Although the contribution of enzymatic steps to total variance was much larger as compared with fragment separation, the use of capillary electrophoresis as compared with flatbed electrophoresis still improved the total variance significantly (median CVs of 26 *versus* 40%).

### 3.3 Signal recognition and matching

When cDNA-AFLP or mRNA differential display is used as a gene discovery tool, gel images or virtual gels generated from electropherograms have often been scored manually in a qualitative fashion. For quantitative analysis of gene expression attempting at coverage of 10^4^–10^5^ signals, signal recognition and matching must be automatized. Wrongly matched signals cause the most serious errors in electrophoresis-based transcriptomics because they may lead to wrong assignments of transcripts to categories “induced,” “suppressed,” and “unaffected” and distort the estimates of relative intensities (induction or suppression factors). Signal matching is therefore a crucial step in data processing.

A major cause of errors in automatic signal matching is that signals of low intensity might be recognized in some but not all lanes or capillaries. Re-analysing the data with a second intensity threshold might be used to reduce the fraction of signals wrongly labeled as solitary. The second crucial parameter affecting the quality of signal matching is PT, which defines the maximum distance allowed for matched signals (in terms of relative mobility or DNA fragment length). We analyzed the effect of intensity threshold on signal recognition and PT on signal matching, monitored the frequency of wrongly matched and unmatched signals, and calculated the variance of intensity for matched signals. Replicates were generated from a single RNA sample as described in Fig. 4.

The first round of signal recognition was started with the intensity threshold of 1% of the intensity of the most intense signal in a lane or capillary and a PT of 0.3%. Two signal-matching correction strategies were than applied. According to the strategy dubbed as “low threshold,” a second signal recognition was performed with zero intensity threshold. Signals remaining unmatched after the first round were matched to the newly recognized signals, and matches satisfying the PT criterion were added to the set of matched signals. According to a second strategy, dubbed “high threshold,” a second signal recognition was started with an intensity threshold of 1% of the total intensity of all signals in a lane or capillary. Signals unmatched after the first round and not recognized by the second round were discarded.

**Figure 6:**
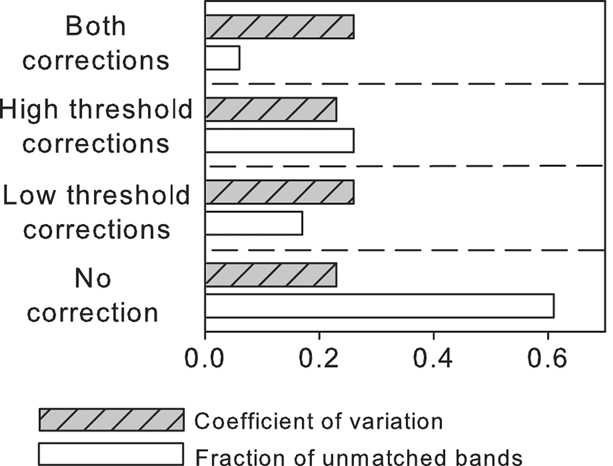
Errors of signal matching. Technical triplicates of cDNA-AFLP products were separated by capillary electrophoresis (CEQ 8000), and raw data were processed with the intensity threshold of 1% of the highest peak in a lane (see Section 2). Two strategies were applied to maximize the fraction of matched signals. According to the “low threshold” strategy, a second round of signal recognition was performed with an intensity threshold of 0%, signals unmatched after the first round were compared with the newly recognized signals, and matches satisfying the PT criterion were added to the set of matched signals. According to “high threshold” strategy, unmatched signals with intensities lower than 1% of the sum of all signals in a lane were discarded. When both strategies were applied, the order was “low threshold” followed by “high threshold”.

The results of these matching improvement strategies are shown in Fig. 6. The fraction of unmatched signals was unacceptably high when no correction was applied. The application of the “low-threshold” strategy, which extended matching to low-intensity signals, reduced the fraction of unmatched signals to 17%. The application of the “high threshold,” which discarded unmatched signals with intensities below a more restrictive threshold, reduced the fraction of unmatched signals to 26%. The combination of both strategies (“high threshold” following “low threshold”) reduced the fraction of unmatched signals to 6%.

The drawback of strategies aimed at the improvement of signal matching is that matches to noise may be generated (“low threshold”) and transcripts strongly suppressed by the treatment as well as inducible transcripts present at very low levels in controls may be lost (“high threshold”). These errors have in common that they pertain to transcripts occurring in very low levels in either controls or treatments. Low-level transcripts, however, are known to cause difficulties in all transcriptomic techniques. They remain undetected or their quantification is inaccurate in microarray hybridization, expressed sequence tag analysis, as well as in serial analysis of gene expression 26.

Is 6% unmatched signals an acceptably low level? At least three replicates are usually processed in transcriptome analysis. At the level of 6% unmatched signals *per* experiment, essentially all signals originating from random artifacts such as noise and electronic spikes will be eliminated by comparing the replicates. Unmatched signals occurring in all replicates, however, will indicate strong induction or suppression.

Although band or peak recognition is primarily controlled by the intensity threshold, the main parameter affecting signal matching is PT. We investigated the effect of PT on matching success and variance of our data in a range from 0.05 to 0.70%, which corresponds to the tolerance of 1 bp for fragments from 143 to 2000 bp (see Section 2).

**Figure 7:**
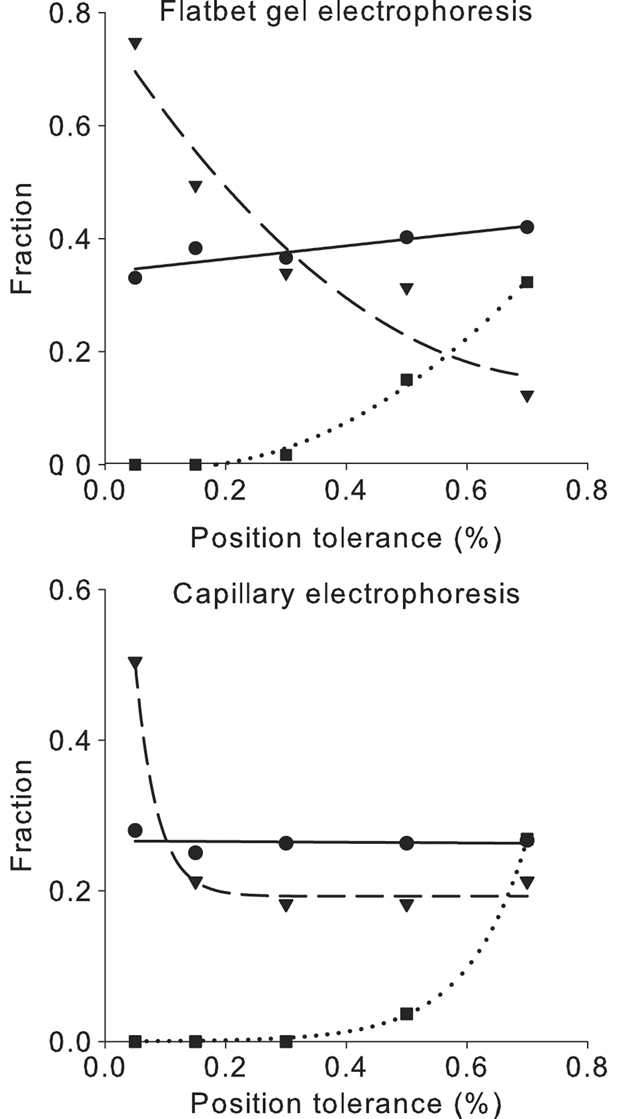
Position tolerance, errors, and variance of signal-matching. cDNA-AFLP products originating from technical triplicates were separated by flatbed gel electrophoresis (ALFExpress II) and capillary electrophoresis (CEQ 8000). The fraction of unmatched signals (triangle with dashed lines), wrongly matched signals (squares with dotted lines), and the variance in intensity-normalized values for matched signals (dots with filled lines) were plotted against PT values ranging from 0.05 to 0.70%.

Figure 7 shows the effect of PT on matching errors and variance for the results of a cDNA-AFLP experiment performed with a flatbed sequencer and a capillary DNA sequencer. The fraction of unmatched signals is high because none of the correction strategies described above was applied. The fraction of unmatched signals decreased in both data sets with increasing PT as expected, but the character of this improvement differed between the electrophoretic systems. For the flatbed sequencer, the fraction of unmatched bands declined through the whole range investigated, whereas for the capillary sequencer a saturation point was reached at PT of 0.2%. Our interpretation of this difference is that, on the capillary sequencer at PT higher than 0.2%, missed matching was not caused by differences in the electrophoretic behavior of capillaries. Peaks remaining unmatched at higher PT may have originated from noise, detector artifacts, or stochastic phenomena leading to band losses. On the flatbed sequencer, increasing PT over the whole range up to 0.7% continued to improve the band-matching score, indicating that differences in normalized mobilities between gel lanes were larger than 0.7%. Reproducibility was better with electrophoresis in capillaries than in flat gels. The fractions of wrongly matched signals increased in both data sets as PT increased over the whole range tested. To minimize the fraction of unmatched bands as well as wrongly matched bands, we recommend using a PT of 0.3%, which corresponds to a difference of less than 1 bp in fragments up to 450 bp for both electrophoretic systems. At this PT value, we detected 1.7% wrongly matched bands after flatbed electrophoresis and no wrongly matched peak in capillary electrophoresis.

As the ratio of signal intensities for wrongly matched signals is unpredictable, we expected that wrong matches would dramatically increase variance. To quantify this effect, we estimated the statistical variance of matched data as the median of the CV of normalized intensities for bands matched among three replicates. Although high PT values increased the fraction of wrongly matched bands (Fig. 7), the effect on variance was insignificant.

## 4 Concluding remarks

The mean variance of cDNA-AFLP as a prototype of electrophoresis-based transcriptomics is comparable to the variance of microarray hybridization. In contrast to microarrays, however, the variance of cDNA-AFLP is independent of signal intensity. The largest contribution to the variance stems from the preamplification step. Computer-aided signal matching can be significantly improved by combining two passages with different intensity thresholds for band recognition. Matching peaks generated by capillary electrophoresis is less erroneous than matching bands visualized on flatbed electrophoresis gels.

## Abbreviations

AFLP: amplified fragment length polymorphism
PT: position tolerance
